# Community-based management of arterial hypertension and cardiovascular risk factors by lay village health workers for people with controlled and uncontrolled blood pressure in rural Lesotho: joint protocol for two cluster-randomized trials within the ComBaCaL cohort study (ComBaCaL aHT Twic 1 and ComBaCaL aHT TwiC 2)

**DOI:** 10.1186/s13063-024-08226-2

**Published:** 2024-06-06

**Authors:** Felix Gerber, Ravi Gupta, Thabo Ishmael Lejone, Thesar Tahirsylaj, Tristan Lee, Giuliana Sanchez-Samaniego, Maurus Kohler, Maria-Inés Haldemann, Fabian Raeber, Mamakhala Chitja, Malebona Mathulise, Thuso Kabi, Mosoetsi Mokaeane, Malehloa Maphenchane, Manthabiseng Molulela, Makhebe Khomolishoele, Mota Mota, Sesale Masike, Matumaole Bane, Mamoronts’ane Pauline Sematle, Retselisitsoe Makabateng, Madavida Mphunyane, Sejojo Phaaroe, Dave Brian Basler, Kevin Kindler, Thilo Burkard, Matthias Briel, Frédérique Chammartin, Niklaus Daniel Labhardt, Alain Amstutz

**Affiliations:** 1 Division of Clinical Epidemiology, Department of Clinical Research, University Hospital Basel, Basel, Switzerland; 2 University of Basel, Basel, Switzerland; 3 Swiss Tropical and Public Health Institute, Allschwil, Switzerland; 4 SolidarMed Lesotho, Maseru, Lesotho; 5 Ministry of Health Lesotho, Maseru, Lesotho; 6 Faculty of Business, Economics and Informatics, University of Zurich, Zurich, Switzerland; 7 Medical Outpatient Department and Hypertension Clinic, ESH Hypertension Centre of Excellence, University Hospital Basel, Basel, Switzerland; 8 Department of Cardiology, University Hospital Basel, Basel, Switzerland; 9 Department of Health Research Methods, Evidence, and Impact, McMaster University, Hamilton, Canada; 10 Oslo Center for Biostatistics and Epidemiology, Oslo University Hospital, University of Oslo, Oslo, Norway; 11 Bristol Medical School, University of Bristol, Bristol, UK

**Keywords:** Arterial hypertension, Community-based care, Village Health Workers, Community health worker, Clinical decision support system, Non-communicable diseases, Trials within cohort, Africa, Lesotho

## Abstract

**Background:**

Arterial hypertension (aHT) is a major cause for premature morbidity and mortality. Control rates remain poor, especially in low- and middle-income countries. Task-shifting to lay village health workers (VHWs) and the use of digital clinical decision support systems may help to overcome the current aHT care cascade gaps. However, evidence on the effectiveness of comprehensive VHW-led aHT care models, in which VHWs provide antihypertensive drug treatment and manage cardiovascular risk factors is scarce.

**Methods:**

Using the trials within the cohort (TwiCs) design, we are assessing the effectiveness of VHW-led aHT and cardiovascular risk management in two 1:1 cluster-randomized trials nested within the Community-Based chronic disease Care Lesotho (ComBaCaL) cohort study (NCT05596773). The ComBaCaL cohort study is maintained by trained VHWs and includes the consenting inhabitants of 103 randomly selected villages in rural Lesotho. After community-based aHT screening, adult, non-pregnant ComBaCaL cohort participants with uncontrolled aHT (blood pressure (BP) ≥ 140/90 mmHg) are enrolled in the aHT TwiC 1 and those with controlled aHT (BP < 140/90 mmHg) in the aHT TwiC 2. In intervention villages, VHWs offer lifestyle counseling, basic guideline-directed antihypertensive, lipid-lowering, and antiplatelet treatment supported by a tablet-based decision support application to eligible participants. In control villages, participants are referred to a health facility for therapeutic management. The primary endpoint for both TwiCs is the proportion of participants with controlled BP levels (< 140/90 mmHg) 12 months after enrolment. We hypothesize that the intervention is superior regarding BP control rates in participants with uncontrolled BP (aHT TwiC 1) and non-inferior in participants with controlled BP at baseline (aHT TwiC 2).

**Discussion:**

The TwiCs were launched on September 08, 2023. On May 20, 2024, 697 and 750 participants were enrolled in TwiC 1 and TwiC 2. To our knowledge, these TwiCs are the first trials to assess task-shifting of aHT care to VHWs at the community level, including the prescription of basic antihypertensive, lipid-lowering, and antiplatelet medication in Africa. The ComBaCaL cohort and nested TwiCs are operating within the routine VHW program and countries with similar community health worker programs may benefit from the findings.

**Trial registration:**

ClinicalTrials.gov NCT05684055. Registered on January 04, 2023.

## Introduction

Globally, arterial hypertension (aHT) is the single most important risk factor for premature mortality, accounting for 10.8 million or almost 20% of all deaths in 2019 [1, 2]. Low- and middle-income countries (LMICs) bear a disproportionate and growing share of the burden of aHT, while many high-income countries have managed to reduce the burden of aHT substantially over the past decades [1, 3–5]. Besides changes in demography and lifestyle, insufficient preventive and therapeutic health system responses are the main drivers for the disproportionally high cardiovascular morbidity and mortality in LMICs. This results in massive treatment gaps, with less than 20% of people living with aHT in LMICs reaching treatment targets [6–8]. Through improved access to antihypertensive medication alone, about 40 million deaths could be prevented over the next 25 years [9]. The involvement of VHWs may be an effective and sustainable approach to enhance access to aHT care and VHW-delivered aHT screening, monitoring, education, and lifestyle counseling has been assessed in various settings with positive effects on awareness, treatment adherence, cardiovascular risk, and blood pressure (BP) control rates [10–13]. However, compared to community-based interventions by healthcare professionals, the effect of community-based VHW-led interventions on BP reduction was modest, probably because most of the VHW-led interventions did not include pharmacological treatment components [13].

Digital clinical decision support systems (CDSS) [14–17] and simple, evidence-based treatment protocols using antihypertensive single-pill combinations (SPCs) are promising facilitators for a sustainable task-shifting of more comprehensive services to lay VHWs [18, 19]. However, it remains unclear how the use of CDSSs and antihypertensive SPCs can be included safely and effectively in community-based VHW-led aHT care, complementing the screening, counseling, and referral services for which benefit has already been shown [20, 21].

We assess the effectiveness of a VHW-led, CDSS-assisted aHT care model in rural Lesotho in two cluster-randomized trials nested within the ComBaCaL (Community-Based chronic disease Care Lesotho) cohort study (NCT05596773). The ComBaCaL cohort is maintained by local, trained, and supervised VHWs and is a platform for the investigation of chronic diseases and their management in rural Lesotho. The intervention described here has been developed based on a local non-communicable diseases prevalence survey and burden assessment [22, 23], a scoping literature review [24], multiple workshops with different stakeholders in Lesotho and the ComBaCaL pilot cohort study.

## Methods

### Setting

The ComBaCaL cohort study is conducted in 103 randomly selected villages in the two rural districts, Butha-Buthe and Mokhothlong, in Lesotho, a small, landlocked country surrounded by South Africa. In each ComBaCaL village, a VHW was elected by the inhabitants according to the procedures outlined in the Lesotho Ministry of Health Village Health Program policy [25]. VHWs play an important role in the Lesotho healthcare system by linking the community to facility-based health services. They are effectively contributing to the control of HIV/AIDS, especially in remote rural areas [25–27]. Lesotho is a typical example of an African LMIC where a developing health system is facing the double burden of still highly prevalent infectious diseases (HIV/AIDS and tuberculosis) in combination with a rapidly growing non-communicable disease epidemic [23, 28–30].

### Design and hypothesis

**Fig. 1:**
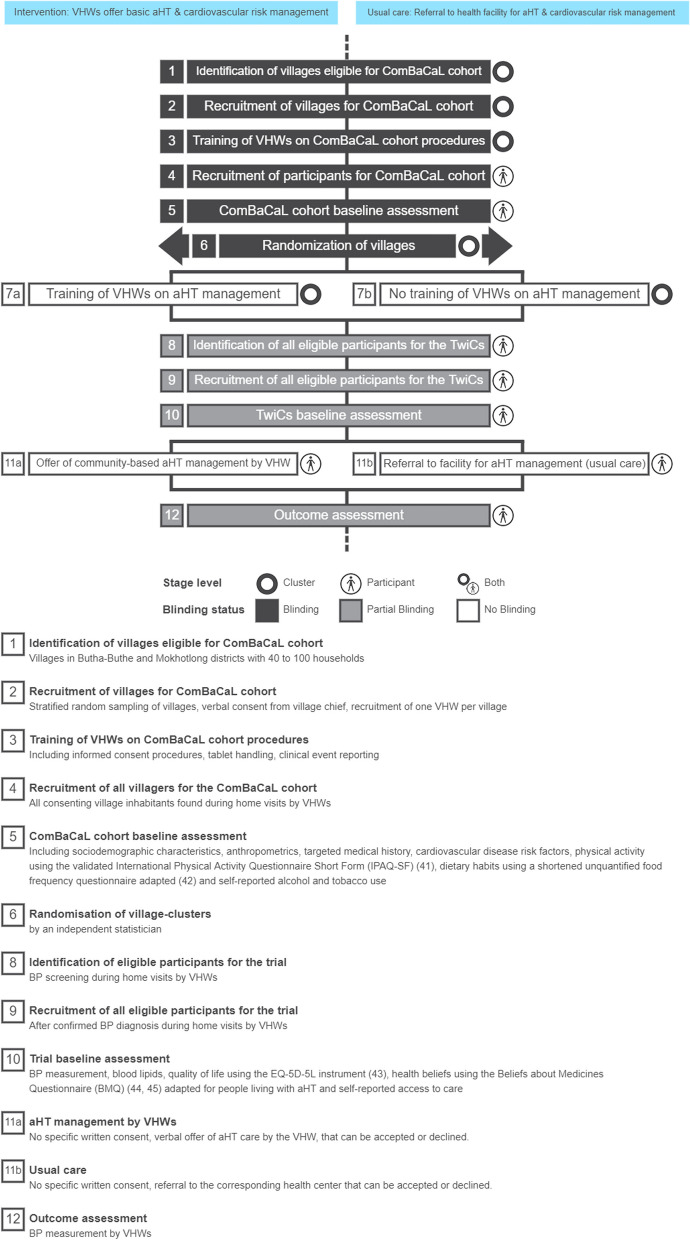
Overview of the ComBaCaL cohort and aHT TwiCs 1 and 2 using the Cluster Timeline Tool [34]

We are conducting two 1:1 cluster-randomized, open-label trials nested within the ComBaCaL cohort study following the trials within the cohort (TwiCs) design [31, 32]. The Standard Protocol Items: Recommendations for Interventional Trials (SPIRIT) reporting guidelines were used to develop and report this protocol [33]. The two TwiCs (aHT TwiC 1 and aHT TwiC 2) use the same cluster-randomization and the same intervention is applied. However, they test different primary hypotheses in distinct populations. The design and flow of events of the two TwiCs are presented in Fig. 1, using the Cluster Timeline Tool [34].

#### Primary hypothesis/estimand aHT TwiC 1

Offering community-based, VHW-led, CDSS-assisted aHT care in rural Lesotho is superior regarding BP control rates (proportion of participants reaching the treatment target < 140/90 mmHg) 12 months after enrollment compared to offering facility-based aHT care among non-pregnant adults with uncontrolled aHT (BP ≥ 140/90 mmHg) who did not experience a traumatic death and did not become pregnant irrespective of the uptake of the intervention, aHT treatment adherence, and adverse events.

#### Primary hypothesis/estimand aHT TwiC 2

Offering community-based, VHW-led, CDSS-assisted aHT care in rural Lesotho is non-inferior regarding BP control rates 12 months after enrollment compared to offering facility-based aHT care among non-pregnant adults with controlled aHT (BP < 140/90 mmHg) who did not experience a traumatic death and did not become pregnant irrespective of the uptake of the intervention, aHT treatment adherence and adverse events.

### Eligibility and consent procedure

Participants for both TwiCs are recruited among the ComBaCaL cohort population, which includes all consenting inhabitants of the randomly selected ComBaCaL villages. Following the TwiCs design [31, 32], no additional written consent for the TwiCs is asked as the ComBaCaL cohort consent includes consent to being randomized for nested TwiCs assessing low-risk interventions. The participant information materials and the consent forms of the ComBaCaL cohort study (covering the nested TwiCs) are available from the corresponding author on request and on ClinicalTrials.gov (NCT05596773). As per cohort procedures (outlined in the ComBaCaL cohort study protocol available on ClinicalTrials.gov), all adult ComBaCaL cohort participants aged 18 years or older are screened for aHT by their VHW according to the diagnostic algorithm of the Lesotho Standard Treatment Guidelines [35].

All non-pregnant adult ComBaCaL cohort participants with aHT, defined as reporting intake of antihypertensive medication or being newly diagnosed during screening, are eligible for aHT TwiC 1 in case of uncontrolled BP levels (≥ 140/90) or for aHT TwiC 2 in case of controlled BP levels (< 140/90). Participants in the control group are followed according to the standard of care in the ComBaCaL cohort which includes aHT screening and referral to health facility-based care in case of diagnosis. In the intervention group, diagnosed participants are offered the intervention by the VHW which they may accept or refuse in addition to the standard of care in the ComBaCaL cohort.

### Randomization and blinding

Half of the ComBaCaL cohort villages are randomly allocated to the intervention group by a statistician not involved in the study. The random allocation is stratified by district (Butha-Buthe versus Mokhothlong) and access to health facilities (easy versus difficult access, defined as needing to cross a mountain or river or travel > 10 km to the nearest health facility). Due to the cluster-level randomization and TwiCs approach, participants are blinded to the allocation, meaning that participants in the control villages are not aware of the intervention being implemented in other villages. VHWs who are enrolling participants, providing the intervention, and collecting endpoint data are not blinded to the intervention. This partial blinding is reflected in Fig. 1.

### Trial interventions

In control villages, VHWs screen for aHT and confirmed cases are referred to the responsible health facility for further care. VHWs conduct a check-up with another referral (if required) 6 months after enrolment, as part of the routine ComBaCaL cohort care, with no further services provided at the community level.

In intervention villages, VHWs offer a community-based aHT care package that includes lifestyle counseling, prescription of antihypertensive SPCs containing amlopidipine and hydrochlorothiazide at low-dose (5 mg/12.5 mg) or high-dose (10 mg/25 g), lipid-lowering (atorvastatin 10 mg) and antiplatelet (acetylsalicylic acid 100 mg) treatment for eligible participants, and treatment support with regular check-ups for participants who are not reaching sufficient BP control with high-dose antihypertensive SPC or having a contraindication against the offered SPC. Guidance for treatment initiation and titration, drug prescription, counseling, and monitoring is provided via the ComBaCaL app, a specifically developed CDSS based on the current Lesotho Standard Treatment Guidelines [35] in line with international guidelines [36, 37]. All activities conducted by VHWs, including counseling and drug prescription, are captured in the ComBaCaL app. VHWs may request support from supervising study staff or routine health care professionals at the responsible health facility. In case of insufficient BP control under high-dose antihypertensive SPC, unclear diagnosis, potential contraindications, side effects or the presence of clinical alarm signs or symptoms, the ComBaCaL app automatically suggests referral of participants to the health facility. Participants are free to accept or refuse the services offered by the VHW at any time. Participants refusing VHW-led services are referred to the responsible health facility for further management with bi-monthly follow-ups by the VHW at the community level for treatment support.

All VHWs have received a 2-week training on the ComBaCaL cohort procedures including usage of the ComBaCaL app, baseline data collection, reporting of relevant clinical events, and screening and diagnosis of aHT. VHWs in the intervention villages received an additional 2-day training on the aHT intervention.

### Endpoints

The selection of endpoints is based on the International Consortium for Health Outcomes Measurements’ data collection reference guide for hypertension in low- and middle-income countries V 4.0.0 [38]. The primary endpoint is the BP control rate, measured 12 months after enrolment. Secondary and exploratory endpoints are provided in Table 1. For all endpoints measured after 6 months, a window of 150 to 240 days, and for 12 months’ endpoints, a window of 300 to 420 days after enrolment applies.

**Table 1:** Primary, secondary, and exploratory endpoints

Primary endpoint:
• Proportion of participants with controlled (< 140/90 mmHg) blood pressure (BP) 12 months after enrolment
**Secondary endpoints:**
• 10-year risk for a fatal or non-fatal cardiovascular event estimated using the WHO cardiovascular disease risk prediction tool [39] 6 and 12 months after enrolment • Proportion of participants with controlled BP (< 140/90 mmHg) 6 months after enrolment • Mean systolic (SBP) and diastolic (DBP) BP 6 and 12 months after enrolment • CVD risk factors, such as BMI, abdominal circumference, blood lipid status, physical activity using the validated International Physical Activity Questionnaire Short Form (IPAQ-SF) [40], dietary habits using a shortened unquantified food frequency questionnaire adapted from an assessment tool for obesity used in South Africa [41] and alcohol and tobacco use 6 and 12 months after enrolment • Linkage to care: proportion of participants not taking treatment at enrolment who have initiated pharmacological antihypertensive treatment 6 and 12 months after enrolment • Engagement in care: proportion of participants who are engaged in care, defined as reporting intake of antihypertensive medication as per prescription of a healthcare provider (VHW or healthcare professional) 6 and 12 months after enrolment or reaching treatment targets without intake of medication • Occurrence of serious adverse events (SAEs) and adverse events of special interest (AESIs) within 6 and 12 months after enrolment • Self-reported adherence to antihypertensive treatment 6 and 12 months after enrolment
**Exploratory endpoints:**
• Quality of life using the EQ-5D-5L instrument [42] 6 and 12 months after enrolment • Health beliefs using the Beliefs about Medicines Questionnaire adapted for people living with aHT [43, 44] after 12 months • Self-reported access to care and access to medication 6 and 12 months after enrolment • Number of consultations at a health facility and with the VHW within 6 and 12 months after enrolment • Trajectory of participants between facility-based and community-based care in the intervention villages (i.e., number of participants accepting community-based care at baseline, number of people switching to facility-based care and back to community-based care during the study period) • Proportion of participants with aHT who stop drug treatment or interrupt drug treatment for more than 3 weeks or require a switch of drug treatment due to (perceived) adverse events (AEs) within 6 and 12 months after enrolment • Proportion of participants who are reaching treatment targets (BP < 140/90 mmHg) and are reporting no intake of antihypertensive medication after 6 and 12 months • Proportion of eligible participants accessing lipid-lowering medication 6 and 12 months after enrolment • Participants’, VHWs’ and involved health care professionals’ perception of the risks, benefits and problems of community-based management of uncomplicated aHT by VHWs • Causes for the stop or interruption of treatment or switch to health facility-based treatment after initiation by VHWs in the community • Health system costs and individual costs for participants for the management of their condition within the first 6 and 12 months after enrolment • 10-year CVD risk estimated using the Globorisk score [45] and Framingham Risk Score [46] 6 and 12 months after enrolment • Type and dosage of antihypertensive and lipid-lowering medications prescribed by VHWs or healthcare professionals 6 and 12 months after enrolment • Proportion of participants with grade III hypertension (180/110 mmHg) 6 and 12 months after enrolment

*Abbreviations: AESI* adverse event of special interest, *aHT* arterial hypertension, *BMI* body mass index, *BP* blood pressure, *CVD* cardiovascular disease, *DBP* diastolic blood pressure, *SAE* serious adverse event, *SBP* systolic blood pressure, *VHW* village health worker, *WHO* World Health Organization

Adverse events of special interest (AESIs) are defined as adverse events (AEs) consistent with aHT complications, such as stroke, myocardial infarction, hypertensive emergency, new diagnosis of heart failure or chronic kidney disease, and AEs probably related to intake of antihypertensive medication leading to discontinuation of the medication concerned.

### Measurements

Baseline and endpoint assessments are conducted by VHWs guided by the ComBaCaL app through instructions for correct BP measurement procedures, sample collection, and structured questionnaires. BP measurements are conducted according to the Lesotho Standard Treatment Guidelines [35] using automated BP machines (Omron M3 Comfort [HEM7131-E] [47]). BP measurements are taken after determination of the correct cuff size in a sitting position after 5 min of rest with feet on the floor, the arm supported without talking or moving during the measurement. At the first visit, the reference arm is determined by measuring BP on both arms. The arm with higher systolic BP is identified as a reference arm and used for all subsequent BP measurements. The BP value is calculated as the mean of the last two out of three consecutive measurements at intervals of 1 min. For the diagnosis of aHT, two elevated measurements in the range of 140–179/90–109 mmHg on two different days are required or two measurements of 180/110 mmHg or higher on the same day, at least 30 min apart.

Some baseline data are extracted from the ComBaCaL cohort database, including sociodemographic characteristics, anthropometrics, targeted medical history, cardiovascular disease risk factors, physical activity using the validated International Physical Activity Questionnaire Short Form (IPAQ-SF) [40], dietary habits using a shortened unquantified food frequency questionnaire adapted from an assessment tool for obesity used in South Africa [41] and self-reported alcohol and tobacco use (see Fig. 1 and Table 2). After identification of eligible participants for the aHT TwiCs through BP screenings, further trial baseline data are collected including blood lipids, quality of life using the EQ-5D-5L instrument [42], health beliefs using the Beliefs about Medicines Questionnaire adapted for people living with aHT [43, 44] and self-reported access to care.

**Table 2:** Schedule of enrolment, interventions, and assessments according to Standard Protocol Items: Recommendations for Interventional Trials (SPIRIT) [33]

**Timepoint**	** − 400–0** Cohort Baseline	**0** TwiC Baseline	**150–240** 6-month follow-up	**300–420** 12-month follow-up
**ComBaCaL cohort activities:**				
ComBaCaL cohort informed consent^1^	X			
Date of birth	X			
Height, weight, abdominal circumference	X		X	X
Targeted medical history	X			
CVDRFs^2^	X		X	X
T2D Screening	X			
aHT Screening	X			
**TwiC assessments:**				
BP measurements		X	X	X
Health beliefs^3^		X		X
Quality of life^4^		X	X	X
Self-reported access to care and to medication		X	X	X
Blood lipid status^5^		X	X	X
Current antihypertensive and lipid-lowering medication	X	X	X	X
Adherence to antihypertensive medication		X	X	X
Number of health facility visits since enrolment			X	X
Individual level cost assessment			X	X
Screening for relevant clinical events		X	X	X
Screening for clinical alarm signs/symptoms		X	X	X
**TwiC control:**				
Referral to health facility if required		X	X	X
**TwiC intervention:**				
Offer antihypertensive SPC^6^		X	X	X
Offer lipid-lowering and anti-platelet treatment^6^		X	X	X
Provide lifestyle counseling		X	X	X
Provide treatment support^7^		X	X	X
Referral to health facility if required^8^		X	X	X

^1^Including consent to participation in TwiCs

^2^Physical activity using IPAQ-SF [40], dietary habits [41], tobacco and alcohol use

^3^Using the Beliefs about Medicines Questionnaire [43, 44]

^4^Using the EQ-5D-5L instrument [42]

^5^Total cholesterol, LDL, HDL, triglycerides

^6^To participants eligible according to Lesotho Standard Treatment Guidelines [35]

^7^To participants receiving treatment from health facility (i.e., participants using three or more antihypertensive drugs)

^8^In case of insufficient BP control or clinical alarm symptoms

*Abbreviations*: aHT arterial hypertension, *BP* blood pressure, *CVDRF* cardiovascular disease risk factor, *T2D* type 2 diabetes, *TwiC* Trial within Cohort, *LDL* low-density lipoprotein, *HDL* high-density lipoprotein, *SPC* single-pill combination

Endpoint assessments through home visits by VHWs are scheduled 6 months (range 150 to 240 days) and 12 months (300 to 420 days) after TwiC enrolment. During follow-up visits, VHWs in both groups inquire about the occurrence of possible SAEs or AESIs and document them in the ComBaCaL app. In addition, VHWs may solicit AESIs and SAEs through reporting by participants, friends or relatives, screening of participants’ “bukanas” (personal health booklet) and reporting by routine health facility staff any time during the follow-up period.

Possible AESIs and SAEs flagged by the VHWs will be followed up by supervising study staff to collect further clinical information (see Table 2). The pseudonymized reports will be submitted to the study physician who will remain blinded to the allocation. The study physician will classify the reports as SAEs, AESIs, or neither of the two and conduct a causality assessment for events classified as SAEs or AESIs. In addition, questionnaires about participants’ satisfaction with and acceptability of the TwiCs intervention will be administered and semi-structured interviews conducted with a subsample of participants, VHWs, and involved health care professionals to qualitatively explore perceived risks, benefits, problems, and acceptability of community-based, VHW-led aHT care.

### Sample size

Separate sample size calculations for the two TwiCs were performed with the higher of the two defining the overall sample size. Sample sizes were calculated assuming an individual randomization inflated by a design effect that accounts for variation at cluster level, according to Rotondi and Donner [48]. Based on preliminary results from an non-communicable disease prevalence survey in Lesotho [22], we expect an adult prevalence of aHT in rural Lesotho of 18%. Considering an average cluster size of 100 adult inhabitants, the mean number of inhabitants with aHT is estimated at 18 (10 being controlled and 8 uncontrolled).

For aHT TwiC 1, testing the hypothesis that the intervention will lead to superior BP control rates among people with uncontrolled BP, we assume an acceptance rate of 75% in the intervention group (based on a previous study [49] and findings from the pilot cohort), a probability of BP control of 60% among individuals that accept the intervention and 30% among those refusing the intervention. Hence, we assume an overall BP control rate in the intervention group of 52.5%. We further assume an intra-cluster correlation of 0.015 [49, 50], a mean cluster size of 8 (standard deviation = 5), and a probability of BP control of 35% in the control group [22]. A minimal sample size of 304 (152 in each arm, 19 clusters per study arm) is required to detect superiority with a type I error of 0.025 and a statistical power of 80%.

For aHT TwiC 2, testing the hypothesis that the intervention will lead to non-inferior BP control rates among people with controlled BP at baseline, we assume an intra-cluster correlation of 0.015 [49, 50], a mean cluster size of 10 (standard deviation = 5) and a probability of BP control at 12 months of 80% in both intervention and control group. Based on considerations of clinical relevance, the non-inferiority margin is set to a 10% higher probability of failing to reach the primary outcome of BP control (< 140/90 mmHg) in the intervention compared to the control group. This corresponds to an odds ratio (OR) of reaching the primary endpoint of 0.58 between the intervention and the control groups. This implies a minimal sample size of 780 across 78 clusters (390 in each arm, 39 clusters per study arm) to be able to detect non-inferiority with a type I error of 0.025 and a statistical power of 80%. We inflate the calculated maximal number of clusters among the two TwiCs by 25% to account for uncertainties in the estimates of aHT prevalence, number of ComBaCaL village inhabitants, and the acceptance rate reaching a total number of 98 clusters. Due to operational reasons, we finally decided to recruit in all 103 ComBaCaL cohort villages, for an anticipated sample size of 824 participants with uncontrolled aHT (TwiC 1) and 1030 participants with controlled aHT (TwiC 2). Through repeated home visits by the VHWs, maximum recruitment within these 103 villages will be ensured while adding further villages in case the sample size is not reached is not possible.

### Statistical analysis

Analyses will be performed following the principles for the analysis of cluster-randomized trials in health research as outlined by Donner and Klar [51]. For both TwiCs, we will use a mixed effect logistic regression model adjusted for stratification factors, sex, and age, with a random intercept at the level of the clusters.

Primary hypotheses of aHT TwiC 1 (superiority of the intervention in people with uncontrolled BP at baseline) and aHT TwiC 2 (non-inferiority of the intervention in people with controlled aHT at baseline) will be assessed in an intention-to-treat analysis set, including all participants as randomized except those with pregnancy or traumatic death during the follow-up. Our intervention is the offer of a community-based care package, and our primary analyses focus on assessing its effect in a real-world setting. Full adherence to the offered care package is not expected and therefore no strict per-protocol analyses will be performed.

For analyses of secondary outcomes, we will use mixed effects logistic or linear regression models, depending on the nature of the outcome, without formal testing. Serious adverse events, adverse events of special interest, adherence to antihypertensive treatment, and exploratory outcomes will be analyzed descriptively. Details are outlined in the statistical analysis plan available as a supplementary document.

### Data management and monitoring

All ComBaCaL VHWs received a password-protected tablet with the ComBaCaL app installed. The ComBaCaL app serves as CDSS and data collection tool. It is based on the open source Community Health Toolkit Core Framework, a widely-used, offline-first, open source software toolkit designed for community health systems [52]. Data are synchronized regularly to a secure server hosted at the University Hospital Basel, Switzerland. All shared data exports will be pseudonymized. Data are monitored locally by the VHW supervisors and centrally by the data management team of the University Hospital Basel. The intervention assessed in these TwiCs entails the task-shifting of aHT services according to local and international guidelines. It has a low risk profile and therefore neither the establishment of an external data monitoring committee nor a formal interim analysis is planned.

## Discussion

Many countries in Africa and other LMICs have established VHW systems that are traditionally focusing on maternal and neonatal health and on communicable diseases, especially HIV/AIDS [53]. In recent years, evidence has emerged showing a beneficial effect and high cost-effectiveness of VHW-based models for diseases outside the traditional scope, especially for non-communicable diseases [10, 11, 13, 54]. While VHW-based aHT care models focusing on educational, screening, monitoring, and referral services have shown positive effects on linkage to care, adherence, and BP levels, the effectiveness on BP reduction remained limited, probably due to the inability of VHWs to deliver pharmacological treatment [10, 13, 55]. Recently, two landmark studies in China, Malaysia, and Colombia have shown significant and clinically relevant reductions in BP levels and cardiovascular risk through community-based non-physician healthcare worker-led multifaceted interventions including delivery of pharmacological treatment [56, 57]. However, the generalizability of these findings to the African context is limited. The study in China involved village health doctors with multi-year medical training and considerable clinical experience [57], a cadre that is not existing in most LMICs, especially in Africa. The study in Colombia and Malaysia relied on drug prescription by healthcare professionals and community health workers merely delivered the drugs, an approach that might limit effective scale-up. [58]. According to a recent scoping review, our two TwiCs will be the first to assess a multifaceted, community-based, VHW-led aHT care intervention including pharmacological treatment in Africa [24].

A strength of both TwiCs is their implementation within an established village cohort that is integrated into the local healthcare system. The study team will provide monitoring to ensure participant safety and data quality, but the intervention will be provided by VHWs recruited within the Lesotho Ministry of Health VHW program [25] and all parts of the intervention will be delivered according to the current Lesotho Standard Treatment Guidelines [35]. The TwiCs design allows to assess the intervention under conditions close to real life: participants in control villages are not aware of the intervention in other villages, while participants in intervention villages are being offered the intervention with oral consent, as it would be the case in routine care.

As per international recommendations [37], a holistic approach to cardiovascular disease management is applied; the intervention does not only include BP control measures, but consists of a comprehensive package including lifestyle counseling, lipid-lowering and antiplatelet treatment for those eligible, and the estimated 10-year risk for a cardiovascular event is a secondary outcome of both TwiCs. The two aHT TwiCs are implemented in parallel with another TwiC nested in the ComBaCaL cohort that is assessing a similar healthcare service package delivered by the same VHWs but for type 2 diabetes mellitus [59]. This combination allows us to gather valuable experience and empirical evidence about implementing community-based integrated service delivery for two major non-communicable diseases simultaneously.

The World Health Organization guidelines on digital interventions for health system strengthening are recommending the use of mobile CDSS for health workers at the community level [60]. However, a recent systematic review found that conclusive evidence on the effectiveness of digital health interventions on BP control in Africa is absent and that large randomized controlled trials are required to produce such [61]. These two TwiCs will address this evidence gap.

The two TwiCs have several limitations. First, VHWs in control villages are also conducting BP screenings and offer regular referral services, as part of the routine cohort procedures, which in itself is an intervention and has to be considered when interpreting the results. Second, due to the multifaceted nature of the intervention, conclusions on the effects of individual components will be limited. Third, the randomization for the TwiCs happens before the identification of eligible trial participants, because VHWs in each arm need specific training before they identify the first participant in their village. This design choice brings two challenges: the possibility of empty clusters and the possibility of differential recruitment [62]. Those challenges are minimized by VHWs systematically screening and diagnosing all ComBaCaL cohort participants in their villages during repeated home visits according to predefined algorithms and thus identifying all eligible TwiCs participants in their village irrespective of the trial arm.

In summary, these TwiCs are assessing the effectiveness of a comprehensive, CDSS-supported, VHW-led, setting-adapted, community-based aHT intervention within the existing Lesotho Village Health Program. They will generate the evidence required for the future development of community-based aHT care models in Lesotho and similar settings.

## Trial status

This manuscript is based on the approved study protocol version 1.1, dated February 07, 2023. Recruitment for the TwiCs started on September 08, 2023, and is ongoing. 697 participants with uncontrolled BP (≥ 140/90 mmHg) were enrolled in aHT TwiC 1 and 750 participants with controlled BP (< 140/90 mmHg) were enrolled in aHT TwiC 2 on May 20, 2024, prior to submission of this manuscript. We expect enrolment to be completed in June 2024.

## Data Availability

We will make pseudo-anonymized individual data freely available on a suitable repository, such as zenodo.org, along with publication of the study results. The full protocol as submitted to ethics committees is available on clinicaltrials.gov. Study results will be published in a peer-reviewed journal without the use of professional writers and communicated to local health authorities and community stakeholders. Access to the test environment of the ComBaCaL app is available from the corresponding author upon reasonable request.

## Abbreviations

AESI: Adverse event of special interest
aHT: Arterial hypertension
BMI: Body mass index
BP: Blood pressure
CDSS: Clinical decision support system
ComBaCaL: Community-Based chronic disease Care Lesotho
CVDRF: Cardiovascular disease risk factor
HDL: High-density lipoprotein
LDL: Low-density lipoprotein
LMICs: Low- and middle-income countries
SAE: Serious adverse event
SPC: Single-pill combination
TwiC: Trial within Cohort
VHW: Village health worker
